# Low‐Density Lipoprotein Estimated by Various Equations in Patients With Obesity

**DOI:** 10.1155/jobe/4315375

**Published:** 2026-05-17

**Authors:** Johannes Oswald, Iris Bregulla, Monika Bröder, Raphael Reiter, Julian Eberhardt, Julian Gomahr, Dagmar Schaffler-Schaden

**Affiliations:** ^1^ Institute of General Practice, Family Medicine and Preventive Medicine, Paracelsus Medical Private University, Strubergasse 21, Salzburg, 5020, Austria; ^2^ Center for Public Health and Healthcare Research, Paracelsus Medical Private University, Strubergasse 21, Salzburg, 5020, Austria; ^3^ Medical Nutrition Therapy, University Hospital Salzburg, Müllner Hauptstraße 48, Salzburg, 5020, Austria; ^4^ Department of Geriatric Medicine, Christian-Doppler-Klinik, Paracelsus Medical Private University, Ignaz-Harrer-Straße 79, Salzburg, 5020, Austria; ^5^ First Department of Medicine, Paracelsus Medical Private University, Müllner Hauptstrasse 48, Salzburg, 5020, Austria; ^6^ Department of Pediatrics, Paracelsus Medical Private University, Müllner Hauptstraße 48, Salzburg, 5020, Austria

**Keywords:** apolipoprotein B, calculated LDL cholesterol, diagnostic tests, Friedewald, low-density lipoprotein cholesterol, Martin–Hopkins, meal replacement, Sampson/NIH

## Abstract

**Background and Aims:**

Reducing atherogenic lipoproteins to lower cardiovascular risk is a key objective in obesity treatment. Accurate lipoprotein measurements are essential for determining the need for therapy and guiding its management. To identify the best equation for estimating LDL cholesterol, LDL measured directly (LDL‐D) is compared to LDL calculated using equations developed by Friedewald (LDL‐F), Martin (LDL‐M) and Sampson/NIH (LDL‐S). This study also examines the change of LDL and apolipoprotein B levels during a multidisciplinary obesity treatment programme.

**Methods:**

This retrospective observational study included 308 adult patients with obesity who participated in a single‐centre, multidisciplinary treatment programme using meal replacement (Optifast‐professional). This study employed Pearson’s correlations, repeated‐measures analyses of variance (ANOVA) and a paired *t*‐test and also compared calculated values against desirable bias and total allowable error.

**Results:**

The absolute values of LDL‐M and LDL‐S showed less bias and correlated better with LDL‐D than LDL‐F. All equations significantly underestimated absolute LDL values: LDL‐F (mean −12.43 mg/dL, *p* < 0.001), LDL‐M (mean −10.05 mg/dL, *p* < 0.001) and LDL‐S (mean −9.49 mg/dL, *p* < 0.001). LDL‐F also underestimated the change occurring during the programme (3.81 mg/dL; *p* = 0.017). All equations performed poorly against desirable bias and total allowable error. LDL‐D, LDL‐F, LDL‐M, LDL‐S and ApoB levels all decreased significantly, and these decreases remained significant after excluding patients taking lipid‐lowering medications.

**Conclusions:**

LDL estimation equations systematically underestimated LDL in individuals with obesity. Among these, the Martin equation most accurately captured changes in LDL. Consequently, it appears to be the most suitable estimation method. However, direct measurement should remain the preferred method. The multidisciplinary meal replacement programme significantly reduced LDL cholesterol and ApoB.

## 1. Introduction

The prevalence of overweight and obesity continues to rise in many countries, making obesity one of the most significant public health challenges worldwide. It carries substantial social, economic and health‐related consequences and is associated with reduced life expectancy. As a complex, multifactorial chronic disease, obesity can lead to a wide range of complications affecting nearly every organ system, including cardiovascular disease [1, 2].

Managing elevated levels of low‐density lipoprotein (LDL) cholesterol is an important strategy for both primary and secondary prevention of cardiovascular disease. The global burden and mortality of cardiovascular disease are high. In 2021, an estimated 19.91 million deaths were attributed to cardiovascular disease worldwide [3]. One important goal of weight reduction programmes is the reduction of obesity‐related comorbidities, including dyslipidaemia and elevated risk for cardiovascular disease. It is a well‐established fact that obesity often contributes to dyslipidaemia and that weight reduction improves the lipid profile and other cardiovascular risk factors [4].

LDL can be measured directly or estimated using mathematical equations. The reference method of beta quantification is expensive and laborious and therefore not suitable for clinical practice. Since the 1990s, direct LDL assays have become available in clinical laboratories; however, compared to calculating LDL, they remain more time‐consuming, more costly and not universally accessible. Although it is unclear which approach is currently most common in clinical practice, the literature suggests that calculation by different equations might still be the most frequently used method also due to easy implementation in computational systems [5–8]. The first commonly used equation was the Friedewald equation. It was published in 1972 and is the best‐known formula in clinical practice to this day [6, 7, 9]. However, its accuracy has always been in debate, as it is reduced at lower levels of LDL and elevated levels of triglycerides and in nonfasting patients [10]. This is particularly problematic as recent guidelines recommend very low levels of LDL especially in secondary prevention, which may potentially affect the reliability of monitoring treatment targets [4].

In recent years, many new equations for calculating LDL have been published [7]. The equations most often cited were published by Sampson/NIH and Martin et al. [11, 12] These equations outperformed the Friedewald equation in several studies [10]. However, we are not aware of a validation study including a population of patients undergoing an obesity treatment programme.

Scientific literature provides evidence that intentional weight loss in patients with obesity leads to a decrease in triglyceride and probably also in LDL levels [13, 14]. Research utilizing Optifast‐professional total meal replacement for obesity treatment has shown statistically significant decreases in LDL in several studies [15–19], while other studies have shown a reduction in LDL that was not statistically significant [20–23]. The studies showing statistically significant reductions in LDL generally include larger populations than the studies showing a nonsignificant reduction. The largest of these studies is a multicentre observational study from Bischoff et al., which includes 8296 patients [19].

Essential components of lipoprotein particles are apolipoproteins. Most notably, apolipoprotein B (ApoB) is found in several types of lipoproteins, including LDL. Although LDL is the most commonly used lipoprotein parameter concerning cardiovascular risk assessment, it has been suggested that especially in patients with obesity, ApoB is more accurate in assessing cardiovascular risk [4, 24, 25].

First, this study aimed to identify the most accurate equation for estimating LDL in individuals participating in an obesity management programme, focusing on the lipoprotein most commonly used in clinical practice for cardiovascular risk assessment. To this end, we compared directly measured LDL (LDL‐D) with LDL values calculated using the Friedewald (LDL‐F), Martin (LDL‐M) and Sampson/NIH (LDL‐S) equations to determine which method provides the best estimate in patients with obesity.

Second, this study examined changes in ApoB and LDL levels during the programme to evaluate the effectiveness of the intervention in reducing atherogenic lipoproteins.

## 2. Materials and Methods

### 2.1. Study Population and Data Collection

We conducted a retrospective, observational study of patients enrolled in the Adipomed obesity treatment programme between the years 2018 and 2024. This time period was chosen based on the availability of electronic records. A total of 340 patients participated in the Adipomed programme during this period, which is consistent with sample sizes reported in other studies examining LDL calculation methods [26].

A multidisciplinary, outpatient obesity treatment programme called Adipomed, which incorporates total meal replacement followed by gradual reintroduction of solid foods, has been operating for many years. It encompasses a multidisciplinary treatment, including weekly sessions provided by dietitians, physiotherapists, psychologists and regular medical consultations. The programme uses a low‐calorie diet (Optifast‐professional) for weight reduction in the initial intensive phase. This first phase is followed by a transition and a stabilization phase. The programme is offered in a long version (Optifast 52 programme), lasting one year, and a short version, lasting four months. The long version includes patients with a body mass index (BMI) of at least 35 kg/m^2^ and a low‐calorie diet for 12 weeks, and the short version includes patients with a BMI of at least 30 kg/m^2^ and a low‐calorie diet for 6 weeks [27–29].

As part of the Adipomed programme, laboratory tests are performed before starting and at the end of the programme. LDL tests are currently done either by calculating LDL using the Friedewald equation or by direct measurement. If especially the level of LDL is still elevated at the end of the programme, a specific drug therapy might be recommended to start after the programme to reduce cardiovascular risk.

**FIGURE 1:**
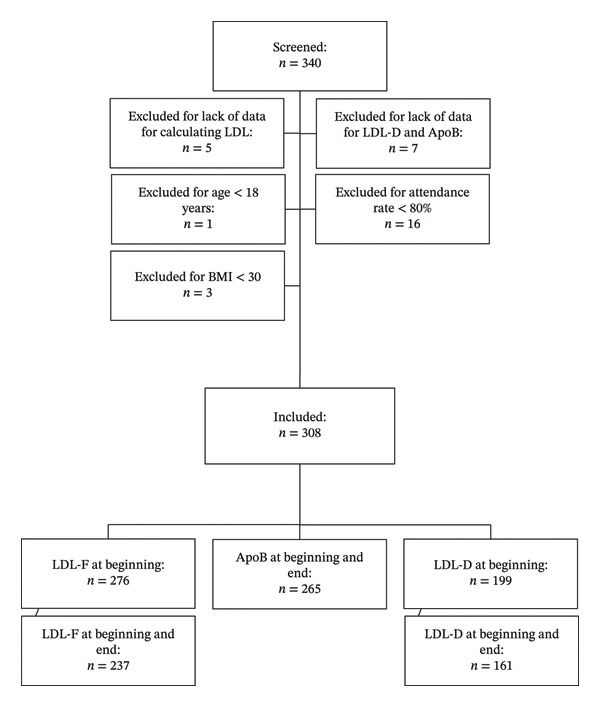
Study population.

In this study, patients from both the long and the short versions of the programme were included without distinction. Most patients lived in the federal state of Salzburg, with a few residing in nearby regions. For patient recruitment, see the flow chart shown in Figure 1, and the characteristics of the study population are provided in Tables 1 and 2. Patients with a Friedewald‐estimated LDL < 70 mg/dL or with triglyceride levels > 375 mg/dL were excluded for analysis of LDL‐F, in accordance with the guidelines of our laboratory. Other patients were excluded for a lack of laboratory results, an attendance rate below 80% during the Adipomed programme, a BMI < 30 kg/m^2^, or age below 18 years, as shown in Figure 1. The enrolment of patients over a period of six years is intended to reduce potential bias due to incomplete laboratory testing.

**TABLE 1:** Further characteristics of the included patients at the beginning and at the end of the Adipomed programme; weight was not recorded at the end of the programme for 19 patients and BMI for 18 patients.

Variable	N	Mean	Median	Standard deviation	Minimum	Maximum
Age at beginning (years)	308	49.02	50.96	13.04	19.99	76.59
Height (cm)	308	169.55	168.25	9.07	149.00	196.00
Weight at beginning (kg)	308	113.86	111.25	23.18	75.60	229.50
Weight at the end (kg)	289	94.26	89.85	19.63	57.40	187.80
Difference of weight (kg)	289	−18.84	−16.00	10.22	−56.50	7.90
Difference of weight (%)	289	−16.42	−15.78	7.42	−38.81	5.77
BMI at beginning	308	39.56	38.38	6.44	30.30	66.30
BMI at the end	290	32.85	31.40	5.71	23.04	58.45
Difference of BMI	290	−6.56	−5.73	3.42	−16.92	2.37
ApoB at the beginning (mg/dL)	303	101.38	99.00	25.65	44.00	179.00
LDL‐D at the beginning (mg/dL)	199	128.80	125.00	40.60	28.00	225.00
LDL‐F at the beginning (mg/dL)	276	122.11	117.30	32.47	38.60	215.00
LDL‐M at the beginning (mg/dL)	308	117.89	115.56	35.97	31.37	212.67
LDL‐S at the beginning (mg/dL)	308	118.06	116.36	37.10	19.53	218.29
Total cholesterol at the beginning (mg/dL)	308	196.20	195.50	41.02	91.00	314.00
HDL at the beginning (mg/dL)	308	53.34	51.00	12.49	28.00	90.00
Triglycerides at the beginning (mg/dL)	308	139.58	127.50	62.76	40.00	437.00
ApoB at the end (mg/dL)	267	91.00	89.00	23.79	45.00	190.00
LDL‐D at the end (mg/dL)	226	116.00	111.00	36.08	29.00	215.00
LDL‐F at the end (mg/dL)	247	115.27	111.60	29.74	36.00	210.00
LDL‐M at the end (mg/dL)	289	107.01	104.80	33.97	29.00	206.74
LDL‐S at the end (mg/dL)	289	108.64	105.62	35.04	27.90	213.34
Total cholesterol at the end (mg/dL)	289	185.10	181.00	39.09	90.00	309.00
HDL at the end (mg/dL)	289	58.48	58.00	11.95	32.00	89.00
Triglycerides at the end (mg/dL)	289	98.86	88.00	48.45	34.00	309.00

*Note:* Minimum refers to the lowest and maximum to the highest value.

**TABLE 2:** Important pre‐existing conditions among included patients.

Pre‐existing condition	N
Diabetes	33
Prediabetes	100
Hyperlipidaemia	166
Hypertension	106
Hyperuricemia	53

The data necessary for this study were collected and stored in digital and print form during the Adipomed programme by the programme team.

### 2.2. Ethical Approval

All patients serving as subjects in the investigation gave written informed consent for the usage of their data for research purposes. The study protocol has been approved by the Ethics Committee of the Federal State of Salzburg (Nr. 1110/2024).

### 2.3. Lipid Profile Analysis

The Department of Laboratory Medicine at the University Hospital Salzburg performed most of the laboratory tests. LDL‐D and ApoB were measured in this laboratory on a Roche COBAS 8000 device (Roche Diagnostics, Rotkreuz, Switzerland) according to the manufacturer’s instructions by homogeneous enzymatic colorimetric assay or immunological turbidimetric assay. However, a small part of laboratory tests was also performed in external laboratories. Fasting blood was collected before enrolment in the Adipomed programme, as well as at the end of the programme.

### 2.4. LDL Estimation

LDL‐F and LDL‐S were calculated using the published equations [9, 11]:

LDL‐F = total cholesterol−high‐density lipoprotein−triglycerides/5

(1) LDL−S=total cholesterol0.948−high density lipoprotein0.971−triglyceride8.56+triglyceride∗non−high densitiy lipoprotein2140−triglycerides216100−9.44

The recently published enhanced Sampson‐NIH equation was not utilized because it incorporates ApoB [30]. In a clinical setting, it is unlikely that ApoB levels would be available, while direct LDL measurements would not.

LDL‐M was calculated using the calculator provided at https://ldlcalculator.com (downloaded 19 December 2024).

### 2.5. Statistical Analysis

Data were collected in Microsoft Excel 2016 and analysed in IBM SPSS Statistics Versions 29 and 30. A *p*‐value < 0.05 was considered statistically significant. Statistical analysis was restricted to complete cases for each analysis.

Correlations between calculated LDL and LDL‐D—both absolute values and total changes during the programme—were assessed using Pearson’s correlation coefficient and visualized with Bland–Altman plots. Mean differences were tested using repeated‐measures analysis of variance (ANOVA) with simple contrasts. For comparisons of absolute values, only baseline laboratory measurements were used. Furthermore, the correlation between total change of ApoB and LDL‐D during the programme was assessed using Pearson’s correlation coefficient. Absolute values were additionally evaluated against desirable bias (5.4%) and total allowable error (11.8%) according to the biological variation estimates listed in the European Federation of Clinical Chemistry and Laboratory Medicine database [31].

Mean differences of absolute LDL values (calculated and directly measured) from baseline to programme end were analysed using repeated‐measures ANOVA with repeated contrasts. Mean differences in ApoB were analysed using a paired *t*‐test. Additional analyses excluded patients taking lipid‐lowering medication before, at the beginning of, or at the end of the programme.

Normality was assumed under the central limit theorem. Sphericity was assessed using Mauchly’s test, and Greenhouse–Geisser correction was applied when the assumption of sphericity was violated.

Finally, we conducted a brief, descriptive subgroup analysis using pairwise case exclusion stratified by sex (male/female), diabetes mellitus status (yes/no) and programme type (long/short).

## 3. Results

### 3.1. Study Population

A total of 340 patients participated in the Adipomed programme between 2018 and 2024 and were screened for eligibility. Overall, 32 patients were excluded from the analysis (see flow chart in Figure 1 for details).

Of 308 patients included in this study, not all laboratory parameters were available at both baseline and programme end (Figure 1), resulting in different sample sizes across analyses. For the repeated‐measures ANOVAs assessing changes in LDL values, 126 patients were included. For the ANOVA assessing absolute baseline LDL values, 175 patients were included.

Among the 308 included patients, 69 were taking lipid‐lowering medication before or during programme participation. Of these, 68 used statins; 10 used ezetimibe, and one patient each used colestyramine, a fibrate or a PCSK9 inhibitor.

The study population consisted of 106 men and 202 women; 187 patients participated in the long version and 121 in the short version of the programme. Thirty‐two screened patients participated twice, 20 were counted as two separate entries, while 12 were included only once due to exclusion criteria applying to one of their programme cycles. Further baseline characteristics are presented in Table 1 and comorbidities in Table 2.

Most laboratory analyses were conducted at the Department of Laboratory Medicine, University Hospital Salzburg. External laboratories performed 4.5% of all tests at baseline and 5.2% at the end of the programme. Because external laboratories always used direct LDL measurements, 7.04% of direct LDL assessments at baseline and 7.08% at the end of the programme were conducted by these external laboratories.

At baseline, one patient showed a triglyceride concentration of 437 mg/dL; all other values were < 400 mg/dL. Therefore, the use of the extended Martin–Hopkins equation was not required [32].

### 3.2. Comparing Absolute Values of LDL‐D, LDL‐F, LDL‐M and LDL‐S

As shown in Table 3 and Figure 2, LDL‐D correlated highly significantly with all three calculated LDL measures. Correlations with LDL‐M (*r* = 0.945, *p* < 0.001) and LDL‐S (*r* = 0.946, *p* < 0.001) were stronger than with LDL‐F (*r* = 0.919, *p* < 0.001). Repeated‐measures ANOVA with Greenhouse–Geisser correction yielded significant overall differences among LDL‐D, LDL‐F, LDL‐M and LDL‐S (F = 104.631; *p* < 0.001). Planned simple contrasts showed statistically significant mean differences relative to LDL‐D: LDL‐F (−12.51 mg/dL, *p* < 0.001), LDL‐M (−10.13 mg/dL, *p* < 0.001) and LDL‐S: (−9.56 mg/dL *p* < 0.001).

**TABLE 3:** Correlation between absolute values of lipoproteins measured before the beginning of the Adipomed programme.

Variable	Test	LDL‐F	LDL‐M	LDL‐S
LDL‐D	Pearson’s correlation	0.919^b^	0.945^b^	0.946^b^
	Sig. (2‐tailed)	< 0.001	< 0.001	< 0.001
	N	175^a^	199	199

^a^Patients with a Friedewald‐estimated LDL < 70 mg/dL or with triglyceride levels > 375 mg/dL were excluded for analysis of LDL‐F.

^b^Correlation is significant at the 0.01 level (2‐tailed).

FIGURE 2Bland–Altman plots between absolute values of lipoproteins measured before the beginning of the Adipomed programme.

**Figure:**
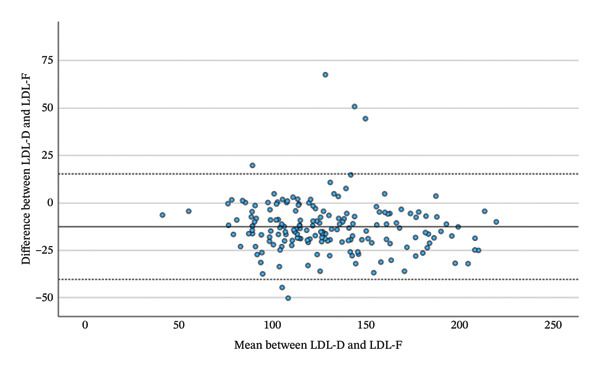
(a)

**Figure:**
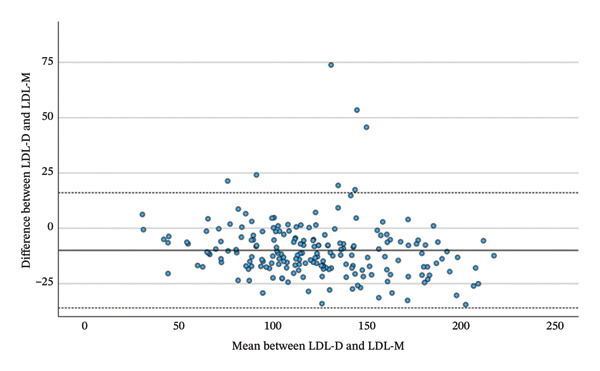
(b)

**Figure:**
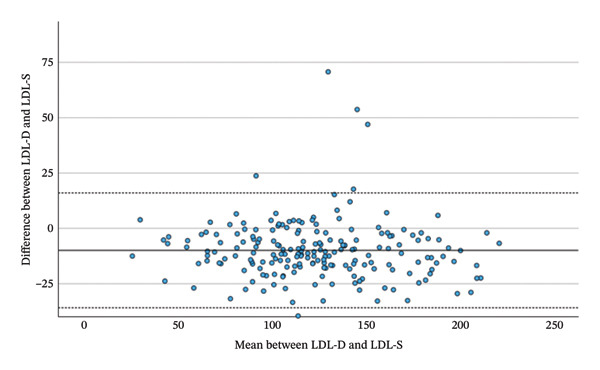
(c)

The proportions of calculated LDL values meeting the desirable bias (5.4%) and total allowable error (11.8%) compared with LDL‐D are presented in Table 4. To provide an estimate of potential undertreatment, Table 5 presents a classification of LDL values into < 116 mg/dL versus ≥ 116 mg/dL. This threshold represents the highest recommended treatment target according to ESC/EAS guidelines, and all patients with LDL levels ≥ 116 mg/dL are therefore clearly above the guideline‐defined target range [4].

**TABLE 4:** Results of calculated LDL with different equations that met the desirable bias and total allowable error compared to LDL‐D.

Variable	Unit	LDL‐F	LDL‐M	LDL‐S
Results within desirable bias (±5.4%)	N	44	51	57
	%	25.14	25.63	28.64
Results within total allowable error (±11.8%)	N	99	120	126
	%	56.57	60.3	63.32
Total amount of results	N	175	199	199

**TABLE 5:** LDL levels of participants at the beginning and the end of the programme categorized into LDL < 116 mg/dL and LDL ≥ 116 mg/dL referring to the highest treatment goal (for low‐risk patients) according to the ESC/EEAS Guidelines for the management of dyslipidaemias [4] (table includes only complete cases, *n* = 126).

	LDL‐D (*n*)	LDL‐D (%)	LDL‐F (*n*)	LDL‐F (%)	LDL‐M (*n*)	LDL‐M (%)	LDL‐S (*n*)	LDL‐S (%)
LDL < 116 mg/dL at the beginning	37	29.4	59	46.8	55	43.65	55	43.65
LDL ≥ 116 mg/dL at the beginning	89	70.6	67	53.2	71	56.35	71	56.35
LDL < 116 mg/dL at the end	58	46.03	69	54.76	71	56.35	65	51.59
LDL ≥ 116 mg/dL at the end	68	53.97	57	45.24	55	43.65	61	48.41

### 3.3. Comparing Changes Between the Beginning and the End of the Adipomed Programme

Comparing the total changes in LDL‐D, LDL‐F, LDL‐M and LDL‐S between the beginning and the end of the Adipomed programme, there is a significant correlation between the changes of calculated parameters and LDL‐D. LDL‐D shows a better correlation with LDL‐M (*r* = 0.869, *p* < 0.001) and LDL‐S (*r* = 0.871, *p* < 0.001) than with LDL‐F (*r* = 0.820, *p* < 0.001), as shown in Table 6 and Figure 3. However, a repeated‐measures ANOVA with Greenhouse–Geisser correction shows a significant difference somewhere between the changes in LDL‐D, LDL‐F, LDL‐M and LDL‐S (F = 4.445; *p* = 0.034). While the mean change in LDL‐D is −10.52 mg/dL, the mean changes in calculated LDL are smaller. A simple contrast comparing calculated values to the change of LDL‐D shows that the change in LDL‐F has a significant difference of 3.72 mg/dL (*p* = 0.019), while the difference in means for the changes of LDL‐M (1.1 mg/dL; *p* = 0.474) and LDL‐S (2.66 mg/dL; *p* = 0.085) is statistically not significant. As this is a comparison between negative numbers, positive differences indicate a smaller change. Finally, a highly significant correlation was observed between the total changes in LDL‐D and ApoB (*r* = 0.830, *p* < 0.001).

**TABLE 6:** Correlation between the change of LDL‐D with the change of LDL‐F, LDL‐M and LDL‐S during the adipomed programme.

Variable	Test	Change LDL‐F	Change LDL‐M	Change LDL‐S
Change of LDL‐D	Pearson’s correlation	0.820^a^	0.869^a^	0.871^a^
	Sig. (2‐tailed)	< 0.001	< 0.001	< 0.001
	N	126	161	161

^a^Correlation is significant at the 0.01 level (2‐tailed).

FIGURE 3Bland–Altman plots between the change of LDL‐D and the change of LDL‐F, LDL‐M and LDL‐S during the adipomed programme.

**Figure:**
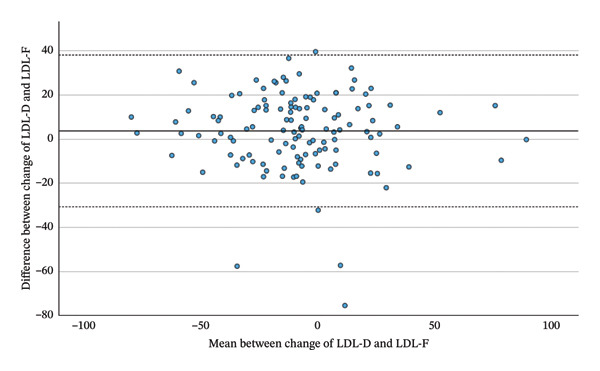
(a)

**Figure:**
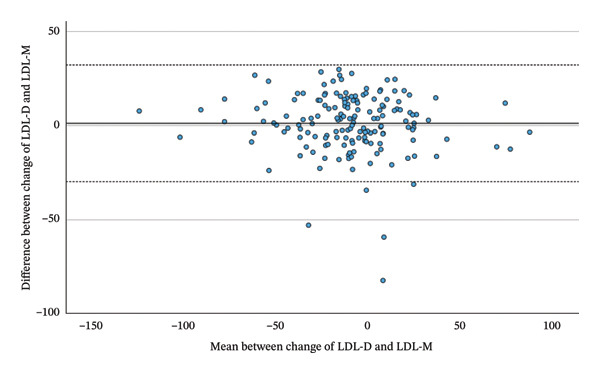
(b)

**Figure:**
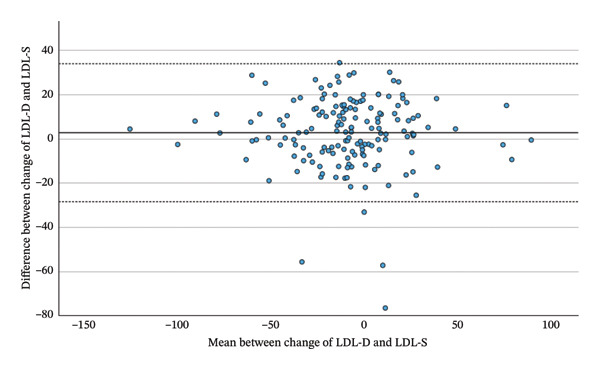
(c)

### 3.4. Difference of Absolute Values Between the Beginning and the End of the Adipomed Programme

Comparing the absolute values of measured and calculated LDL between the beginning and the end of the programme, a repeated‐measures ANOVA with Greenhouse–Geisser correction showed a significant difference (F = 18.843; *p* < 0.001). In planned repeated contrast, there is a significant difference in LDL‐D (mean −10.52 mg/dL; *p* < 0.001), LDL‐F (mean −6.8 mg/dL; *p* = 0.009), LDL‐M (mean −9.42 mg/dL; *p* < 0.001) and LDL‐S (mean −7.85 mg/dL; *p* = 0.003).

When excluding patients with lipid‐lowering medications, a repeated‐measures ANOVA with Greenhouse–Geisser correction also showed a significant difference (*n* = 103; F = 27.474; *p* < 0.001). In planned repeated contrast, there is also a significant difference in LDL‐D (mean −11.82 mg/dL; *p* < 0.001), LDL‐F (mean −7.19 mg/dL; *p* = 0.001), LDL‐M (mean −9.75 mg/dL; *p* < 0.001) and LDL‐S (mean −8.25 mg/dL; *p* < 0.001).

When comparing ApoB at the beginning and end of the programme, the mean difference is −10.23 mg/dL with high statistical significance (*p* < 0.001; *n* = 265). When excluding patients with lipid‐lowering medications, the mean difference is −9.66 also with high statistical significance (*p* < 0.001; *n* = 207).

This study shows a reduction in LDL and ApoB in patients after participating in the Adipomed programme. This reduction is statistically significant for both ApoB and directly measured and calculated LDL, irrespective of which of the three equations is used. These reductions are also significant if patients taking lipid‐lowering medications are excluded from analysis.

### 3.5. Subgroup Analysis

The descriptive subgroup analysis is presented in Figure 4. At baseline, when comparing absolute values of calculated and directly measured LDL, all equations underestimated LDL across the subgroups. A possible exception was observed in patients with diabetes mellitus, among whom only the LDL‐F equation produced a higher mean value than directly measured LDL. In contrast, the subgroup results for LDL changes during the programme were less consistent. Unlike the findings in the overall population, the equations may overestimate LDL change in men and in patients with diabetes mellitus. It is also evident that the impact on lipoprotein levels is greater in the short programme compared with the long programme.

FIGURE 4Subgroup analysis concerning sex, diabetes mellitus and type of programme.

**Figure:**
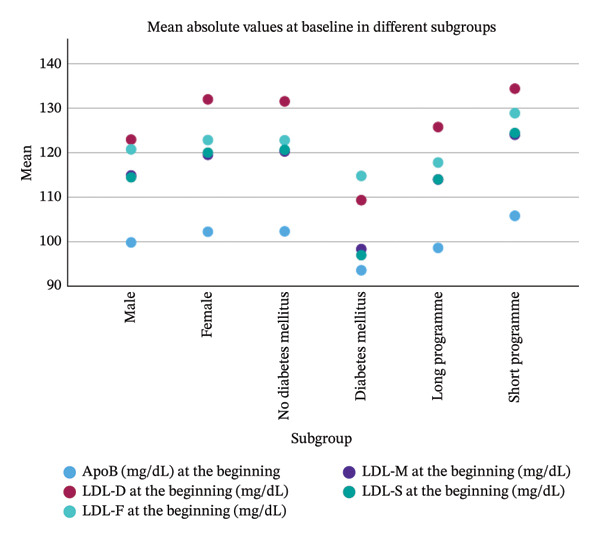
(a)

**Figure:**
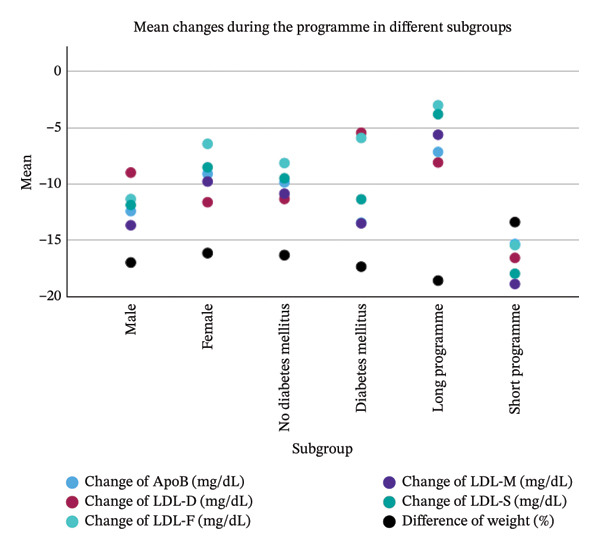
(b)

## 4. Discussion

In summary, looking at absolute values during the Adipomed programme, LDL calculated by the equations of Martin and Sampson/NIH consistently shows less bias and better correlation with directly measured LDL than LDL calculated using the Friedewald equation. However, absolute values are consistently underestimated by all equations compared to direct measurement. This observation remains consistent across the analysed subgroups, specifically those defined by diabetes status, sex and programme type. The Friedewald equation also underestimates the change of LDL during the programme compared to directly measured LDL. The Sampson equation might also underestimate this change, although the difference is not statistically significant in this study. It can be hypothesized that this difference might become statistically significant in a larger population. However, the difference between LDL‐D and LDL‐M regarding the change during the programme is clearly not statistically significant in this population. There might be some concerns regarding men and patients with diabetes looking at the subgroup analysis. However, LDL calculated by the Martin equation seems to be the best choice of the three equations studied, at least to detect changes in LDL during the Adipomed programme.

Analysing the proportion of calculated LDL meeting desirable bias and total allowable error, the equations of Martin and Sampson are clearly performing better than the Friedewald equation. Nevertheless, the percentage of calculated values meeting the desirable bias and total allowable error is low in all equations. However, the percentages are not far off from a recent publication by Paydas Hataysal et al. and therefore seem plausible [33]. Based on the biological variation estimates listed in the European Federation of Clinical Chemistry and Laboratory Medicine database, the use of all three equations might not be recommended for patients with obesity. Based on our findings, direct measurement of LDL (or ApoB for estimating cardiovascular risk) should be the preferred approach [31].

The subgroup analysis also showed a greater reduction in LDL and ApoB during the short programme compared with the long programme. This finding is somewhat unexpected, given that the mean relative weight reduction was higher in the long programme—a plausible result considering its 12‐month duration compared with 4 months for the short programme. As greater weight reduction is generally associated with larger decreases in LDL, this finding appears to contradict previous studies reporting a positive association between weight loss and LDL reduction [13]. However, Zomer et al. observed a similar pattern: In their subgroup analysis of weight loss interventions achieving more than 5% weight reduction, LDL decreases were greater in shorter programmes (6 to < 12 months) than in longer programmes (> 12 months) [34]. One possible explanation is that individuals undergoing a very‐low‐calorie diet may experience a rebound increase in lipoproteins following an initial decline [35]. In the Adipomed programme, after the initial total meal replacement phase, regular food is gradually reintroduced. This reintroduction phase is longer in the long programme than in the short programme, meaning that the postintensive‐phase rise in lipoproteins may be further progressed by the end of the long programme than by the end of the short programme.

Many studies in the literature have shown an underestimation of absolute LDL results calculated using the Friedewald equation compared to direct measurement results. The Martin and Sampson/NIH equations show superior accuracy compared to the Friedewald equation, but whether they tend to under‐ or overestimate LDL is not consistent across published studies [7, 26, 36–38]. This inconsistency might arise due to different study populations and different methods for direct measurement of LDL [39].

Our study provides further evidence that LDL is reduced by a low‐calorie diet using Optifast‐professional. It shows a statistically significant reduction in LDL irrespective of whether LDL is measured directly or calculated by different equations. Except for the Friedewald equation, the reduction of LDL is statistically highly significant (*p* ≤ 0.01).

However, focusing only on blood LDL levels might be misleading when estimating cardiovascular risk, especially in patients without substantially elevated LDL levels. This is because obesity may lead to a reduced LDL particle size, which has a higher atherogenic effect [14]. But a reduction in cardiovascular risk appears plausible, as ApoB levels also declined significantly and showed a strong, highly significant correlation with the decrease in LDL‐D. This finding is in accordance with Gossain et al., who showed a statistically significant reduction in ApoB using Optifast‐professional [22], and Shoji et al., who also showed a reduction in ApoB, although not statistically significant and in a very small study population [23].

In clinical practice, the decision for a lipid‐lowering therapy is still mostly made based on LDL levels. For Adipomed patients, this decision is typically made after completing the programme in a ‘treat obesity first’ approach, as also recently recommended by the American Obesity Medicine Association [40]. There is always hope that the lipoproteins of a patient might be sufficiently reduced by successfully completing the programme, thus avoiding the need for pharmacological treatment. The underestimation of LDL levels shown in this study between LDL‐D and calculated LDL could carry meaningful clinical implications, as illustrated using the 116 mg/dL cut‐off. At this threshold, a notable proportion of patients is misclassified at both the beginning and the end of the programme.

Concerning patients with obesity, this could lead to undertreatment with lipoprotein‐lowering drugs. Although ApoB or non‐HDL would be more accurate parameters for cardiovascular risk assessment in patients with obesity, in clinical practice, LDL is still in widespread use [14]. Because the decision to initiate lipid‐lowering pharmacotherapy is usually postponed until the programme has been completed, patients enrolled in a multidisciplinary obesity programme may miss the opportunity to achieve the optimal cardiovascular risk‐reduction benefit during the course of the intervention.

Our study provides evidence that all three equations tend to underestimate LDL in a population of adults with obesity. This is particularly important because the measurement of LDL already tends to underestimate exposure to pro‐atherogenic lipoproteins in patients with obesity [4].

## 5. Limitations

This study has several strengths, such as the exclusion of patients taking lipid‐lowering medications when comparing lipoprotein levels before and after the treatment programme, the timespan of 6 years during which all patients taking part in the programme were screened or the population of exclusively obese patients with a BMI of > 30 at the beginning of the programme. However, it also has several limitations. One limitation is that it is a retrospective, single‐centre study, although the sample size seems representative. The laboratory measurements were performed by independent laboratories using different methods for direct measurement of LDL. This aspect serves as both a strength and a limitation of the study. On the one hand, having various laboratories involved may lead to less standardized measurements of LDL‐D. On the other hand, it enhances the generalizability of the results. Direct measurement of LDL also has known inaccuracies, such as differences between measurement systems and inaccuracy in patients with high levels of triglycerides [4, 39]. However, triglyceride levels of our population are not extremely high. Additionally, the measurement of different laboratory parameters was not consistently done for all Adipomed patients, which is the reason why different numbers of patients were included in different analyses. There were different laboratory testing panels used over the years. Because not all comorbidities were accounted for during data collection, it was not possible to perform an individual cardiovascular risk assessment to determine patient‐specific LDL target levels. Finally, due to the resulting limited number of included patients, a sex‐ and gender‐based analysis was not performed.

## 6. Conclusions

In this population, the equations for estimating LDL—Friedewald, Martin and Sampson/NIH—underestimate the absolute values and, except for the Martin equation, also the changes during the Adipomed programme. Additionally, they largely fail to meet the desirable bias and total allowable error standards when compared to directly measured LDL. This could lead primarily to undertreatment of patients with obesity with lipoprotein‐lowering drugs. In conclusion, direct measurement of LDL or ApoB should be used to assess cardiovascular risk and make therapy decisions in patients with obesity. Only if LDL must be calculated, the Martin equation seems to be the preferred method. Furthermore, this study provides evidence that LDL and ApoB are reduced in adult patients with obesity through a multidisciplinary obesity treatment programme that includes meal replacement using Optifast‐professional.

**Nomenclature**
AI: artificial intelligence
ANOVA: analysis of variance
ApoB: apolipoprotein B
BMI: body mass index
cm: centimetres
HDL: high‐density lipoprotein
kg: kilograms
LDL: low‐density lipoprotein cholesterol
LDL‐D: LDL measured directly
LDL‐F: LDL calculated by the Friedewald equation
LDL‐M: LDL calculated by the Martin equation
LDL‐S: LDL calculated by the Sampson/NIH equation
mg/dL: milligrams per decilitre
NIH: National Institutes of Health

## Author Contributions

Johannes Oswald: conceptualization, methodology, investigation, data curation, formal analysis and writing–original draft. Iris Bregulla: investigation, data curation and writing–review and editing. Monika Bröder: resources, data curation and writing–review and editing. Raphael Reiter: writing–review and editing. Julian Eberhardt: writing–review and editing. Julian Gomahr: writing–review and editing. Dagmar Schaffler‐Schaden: project administration, funding acquisition, investigation, data curation and writing–review and editing.

## Funding

This work is a part of the ‘Management of Obesity in Salzburg (OBMAN)’ project and was supported by the Research and Innovation Fund of the Paracelsus Medical Private University Salzburg (grant number 2023‐liF‐011‐Schaffler‐Schaden).

Open access funding was provided by Paracelsus Medizinische Privatuniversität/KEMÖ.

## Disclosure

All authors critically reviewed and approved the final manuscript.

## Conflicts of Interest

Monika Bröder received travel reimbursements from Nestlé, which were not directly related to the Adipomed programme or Optifast. Nestlé had no impact on the results or outcomes of this study. The other authors declare no conflicts of interest.

## Data Availability

The data that support the findings of this study are available from the corresponding author upon reasonable request.

## References

[1] OECD , The Heavy Burden of Obesity: The Economics of Prevention, 2019, OECD, 10.1787/67450d67-en.

[2] Kinlen D. , Cody D. , and O’Shea D. , Complications of Obesity, QJM: International Journal of Medicine. (2018) 111, no. 7, 437–443, 10.1093/qjmed/hcx152, 2-s2.0-85048566568. 29025162

[3] Martin S. S. , Aday A. W. , Almarzooq Z. I. et al., On Behalf of the American Heart Association Council on Epidemiology and Prevention Statistics Committee and Stroke Statistics Subcommittee, 2024 Heart Disease and Stroke Statistics: A Report of US and Global Data From the American Heart Association, Circulation. (2024) 149, no. 8, 10.1161/CIR.0000000000001209. PMC12146881 38264914

[4] Mach F. , Baigent C. , Catapano A. L. et al., ESC/EAS Guidelines for the Management of Dyslipidaemias: Lipid Modification to Reduce Cardiovascular Risk: The Task Force for the Management of Dyslipidaemias of the European Society of Cardiology (ESC) and European Atherosclerosis Society (EAS), European Heart Journal. (2019) 41, no. 1, 111–188, 10.1093/eurheartj/ehz455. 31504418

[5] Piani F. , Cicero A. F. G. , Borghi C. , and D’Addato S. , Is the 2020 Sampson Equation the Best Formula for LDL-C Estimation?, European Journal of Internal Medicine. (2021) 83, 99–101, 10.1016/j.ejim.2020.09.009. 32978038

[6] Mănescu I. B. , Demian L. , and Dobreanu M. , Low-Density Lipoprotein Cholesterol Gymnastics: Exploring the Advantages and Limitations of the Friedewald, Martin–Hopkins, and Sampson Equations for Personalized Lipid Management, Journal of Physical Mathematics. (2024) 14, no. 9, 10.3390/jpm14091000. PMC11433184 39338254

[7] Samuel C. , Park J. , Sajja A. et al., Accuracy of 23 Equations for Estimating LDL Cholesterol in a Clinical Laboratory Database of 5,051,467 Patients, Global Heart. (2023) 18, no. 1, 10.5334/gh.1214. PMC10289049 37361322

[8] Wolska A. and Remaley A. T. , Measuring LDL-Cholesterol: What is the Best Way to Do it?, Current Opinion in Cardiology. (2020) 35, no. 4, 405–411, 10.1097/HCO.0000000000000740. 32412961 PMC7360339

[9] Friedewald W. T. , Levy R. I. , and Fredrickson D. S. , Estimation of the Concentration of Low-Density Lipoprotein Cholesterol in Plasma, Without Use of the Preparative Ultracentrifuge, Clinical Chemistry. (1972) 18, no. 6, 499–502, 10.1093/clinchem/18.6.499. 4337382

[10] Martins J. , Steyn N. , Rossouw H. M. , and Pillay T. S. , Best Practice for LDL-Cholesterol: When and How to Calculate, Journal of Clinical Pathology. (2023) 76, no. 3, 145–152, 10.1136/jcp-2022-208480. 36650044

[11] Sampson M. , Ling C. , Sun Q. et al., A New Equation for Calculation of Low-Density Lipoprotein Cholesterol in Patients With Normolipidemia and/or Hypertriglyceridemia, JAMA Cardiology. (2020) 5, 1–9, 10.1001/jamacardio.2020.0013. PMC7240357 32101259

[12] Martin S. S. , Blaha M. J. , Elshazly M. B. et al., Comparison of a Novel Method vs the Friedewald Equation for Estimating Low-Density Lipoprotein Cholesterol Levels From the Standard Lipid Profile, JAMA. (2013) 310, no. 19, 10.1001/jama.2013.280532, 2-s2.0-84887958130. PMC4226221 24240933

[13] Hasan B. , Nayfeh T. , Alzuabi M. et al., Weight Loss and Serum Lipids in Overweight and Obese Adults: A Systematic Review and Meta-Analysis, Journal of Clinical Endocrinology & Metabolism. (2020) 105, no. 12, 3695–3703, 10.1210/clinem/dgaa673. 32954416

[14] Bays H. E. , Kirkpatrick C. F. , Maki K. C. et al., Obesity, Dyslipidemia, and Cardiovascular Disease: A Joint Expert Review From the Obesity Medicine Association and the National Lipid Association 2024, Journal of Clinical Lipidology. (2024) 18, no. 3, e320–e350, 10.1016/j.jacl.2024.04.001. 38664184

[15] Lange U. G. , Moulla Y. , Schütz T. et al., Effectiveness and Tolerability of a Two-Week Hypocaloric Protein-Rich Diet Prior to Obesity Surgery With Two Different Diet Interventions: A Prospective Randomized Trial, Obesity Surgery. (2022) 32, no. 9, 2903–2913, 10.1007/s11695-022-06180-z. 35851679 PMC9392692

[16] Hausmann J. , Waechtershaeuser A. , Behnken I. et al., The Role of Adipokines in the Improvement of Diabetic and Cardiovascular Risk Factors Within a 52-Week Weight-Loss Programme for Obesity, Obesity Research & Clinical Practice. (2019) 13, no. 5, 440–447, 10.1016/j.orcp.2019.09.006, 2-s2.0-85072793909. 31591082

[17] Barnes M. , Goldsworthy U. R. , Cary B. A. , and Hill C. J. , A Diet and Exercise Program to Improve Clinical Outcomes in Patients With Obstructive Sleep Apnea–a Feasibility Study, Journal of Clinical Sleep Medicine. (2009) 05, no. 5, 409–415, 10.5664/jcsm.27594. PMC2762710 19961023

[18] Ard J. D. , Neeland I. J. , Rothberg A. E. et al., The Optifast Total and Partial Meal Replacement Programme Reduces Cardiometabolic Risk in Adults With Obesity: Secondary and Exploratory Analysis of the OPTIWIN Study, Diabetes, Obesity and Metabolism. (2024) 26, no. 3, 950–960, 10.1111/dom.15392. 38073426

[19] Bischoff S. C. , Damms-Machado A. , Betz C. et al., Multicenter Evaluation of an Interdisciplinary 52-Week Weight Loss Program for Obesity With Regard to Body Weight, Comorbidities and Quality of Life—A Prospective Study, International Journal of Obesity. (2012) 36, no. 4, 614–624, 10.1038/ijo.2011.107, 2-s2.0-84859637663. 21673653 PMC3322430

[20] Rothberg A. , Lanham M. , Randolph J. , Fowler C. , Miller N. , and Smith Y. , Feasibility of a Brief, Intensive Weight Loss Intervention to Improve Reproductive Outcomes in Obese, Subfertile Women: A Pilot Study, Fertility and Sterility. (2016) 106, no. 5, 1212–1220, 10.1016/j.fertnstert.2016.06.004, 2-s2.0-84994860378. 27336206 PMC5797426

[21] Alabdali F. , Rueda-Clausen C. F. , Robbins S. , and Sharma A. M. , Efficacy and Safety of Long-Term Low-Calorie Diet in Severely Obese Patients Non-Eligible for Surgery, Clinical Obesity. (2013) 3, no. 3-4, 90–94, 10.1111/cob.12017. 25586530

[22] Gossain V. V. , Gunaga K. P. , Carella M. J. , Bennink M. , Edminster R. R. , and Rovner D. R. , Apolipoproteins in Obesity: Effect of Weight Loss, Journal of Medicine. (1997) 28, no. 3-4, 251–264. 9355029

[23] Shoji T. , Nishizawa Y. , Koyama H. et al., Lipoprotein Metabolism in Normolipidemic Obese Women During Very Low Calorie Diet: Changes in High Density Lipoprotein, Journal of Nutritional Science and Vitaminology. (1991) 37, no. Suppl, S57–S64, 10.3177/jnsv.37.supplement_s57, 2-s2.0-0026276721. 1820446

[24] Cole J. , Zubirán R. , Wolska A. , Jialal I. , and Remaley A. , Use of Apolipoprotein B in the Era of Precision Medicine: Time for a Paradigm Change?, JCM. (2023) 12, no. 17, 10.3390/jcm12175737. PMC10488498 37685804

[25] De Oliveira-Gomes D. , Joshi P. H. , Peterson E. D. et al., Bridging the Gap Between Evidence and Clinical Practice, Circulation. (2024) 150, 62–79, 10.1161/circulationaha.124.068885. 38950110 PMC11219008

[26] Martins J. , Rossouw H. M. , and Pillay T. S. , How Should Low-Density Lipoprotein Cholesterol be Calculated in 2022?, Current Opinion in Lipidology. (2022) 33, no. 4, 237–256, 10.1097/MOL.0000000000000833. 35942811

[27] Information Adipomed Programme Uniklinikum Salzburg 03_2023_65768062, 2025, https://salk.at.

[28] Adipomed, 2026, https://salk.at/campus-lkh/adipomed.

[29] P & Kommunikation , 25 Jahre Erfolgsgeschichte Adipomed an Der UK Für Innere–Landeskliniken Salzburg | Mediencenter, Landeskliniken Salzburg Mediencenter. (2025) https://presse.salk.at/news-25-jahre-erfolgsgeschichte-adipomed-an-der-uk-fuer-innere-medizin-i?id=220839&menueid=21374&l=deutsch.

[30] Coverdell T. C. , Sampson M. , Zubirán R. et al., An Improved Method for Estimating Low LDL-C Based on the Enhanced Sampson-NIH Equation, Lipids in Health and Disease. (2024) 23, no. 1, 10.1186/s12944-024-02018-y. PMC10851542 38331834

[31] EFLM Biological Variation, 2025, https://biologicalvariation.eu/meta_calculations.

[32] Sajja A. , Park J. , Sathiyakumar V. et al., Comparison of Methods to Estimate Low-Density Lipoprotein Cholesterol in Patients With High Triglyceride Levels, JAMA Network Open. (2021) 4, no. 10, 10.1001/jamanetworkopen.2021.28817. PMC8554644 34709388

[33] Paydaş Hataysal E. , Körez M. K. , Yeşildal F. , and İşman F. K. , A Comparative Evaluation of Low-Density Lipoprotein Cholesterol Estimation: Machine Learning Algorithms Versus Various Equations, Clinica Chimica Acta. (2024) 557, 10.1016/j.cca.2024.117853. 38461864

[34] Zomer E. , Gurusamy K. , Leach R. et al., Interventions that Cause Weight Loss and the Impact on Cardiovascular Risk Factors: A Systematic Review and Meta‐Analysis, Obesity Reviews. (2016) 17, no. 10, 1001–1011, 10.1111/obr.12433, 2-s2.0-84986628195. 27324830

[35] Phinney D. , Tang A. B. , Waggoner C. R. , Tezanos-Pinto R. G. , and Davis P. A. , The Transient Hypercholesterolemia of Major Weight Loss, The American Journal of Clinical Nutrition. (1991) 53, no. 6, 1404–1410. 2035468 10.1093/ajcn/53.6.1404

[36] Cesena F. , Friedewald, Martin/Hopkins Ou Sampson/NIH: Qual O Melhor Método Para Estimar O LDL-Colesterol?, Arquivos Brasileiros de Cardiologia. (2022) 119, no. 2, 234–235, 10.36660/abc.20220455. 35946684 PMC9363056

[37] Chung S. , Correlation of Extended Martin/Hopkins Equation With a Direct Homogeneous Assay in Assessing Low‐Density Lipoprotein Cholesterol in Patients With Hypertriglyceridemia, Journal of Clinical Laboratory Analysis. (2023) 37, no. 17-18, 10.1002/jcla.24963. PMC10623527 37679962

[38] Naser A. , Isgandarov K. , Güvenç T. S. , Güvenç R. Ç. , and Şahin M. , Comparação Das Novas Equações De Martin/Hopkins E Sampson Para O Cálculo Do Colesterol De Lipoproteína De Baixa Densidade em Pacientes Diabéticos, Arquivos Brasileiros de Cardiologia. (2022) 10.36660/abc.20210641. PMC9363054 35766617

[39] Ertürk Zararsız G. , Bolat S. , Cephe A. et al., Validation of Friedewald, Martin-Hopkins and Sampson Low-Density Lipoprotein Cholesterol Equations, PLoS One. (2022) 17, no. 5, 10.1371/journal.pone.0263860. PMC9106156 35559957

[40] Bays H. E. , Bindlish S. , and Clayton T. L. , Obesity, Diabetes Mellitus, and Cardiometabolic Risk: An Obesity Medicine Association (OMA) Clinical Practice Statement (CPS) 2023, Obesity Pillars. (2023) 5, 10.1016/j.obpill.2023.100056. PMC10661981 37990743

